# Evolution of resources and study of factors associated with the quality of care for epidemic-prone diseases in Guinea

**DOI:** 10.21203/rs.3.rs-9612015/v1

**Published:** 2026-05-06

**Authors:** DIMAI OUO KPAMY, Fatoumata CHERIF, Sory CONDE, Fodé Amara TRAORE, Sidikiba SIDIBE, Bassirou DIARRA, Peter John WINCH, Abdoulaye TOURE, Sakoba KEITA, Seydou DOUMBIA, Alexandre DELAMOU

**Affiliations:** Faculty of Health Sciences and Technology, Gamal Abdel Nasser University, Conakry, Guinea; National Agency for Health Security, Conakry, Guinea; National Agency for Health Security, Conakry, Guinea; Faculty of Health Sciences and Technology, Gamal Abdel Nasser University, Conakry, Guinea; Faculty of Health Sciences and Technology, Gamal Abdel Nasser University of Conakry; University of Sciences, Techniques and Technologies of Bamako (USTTB); Department of International Health, Johns Hopkins Bloomberg School of Public Health, Baltimore, MD, United States; Faculty of Health Sciences and Technology, Gamal Abdel Nasser University of Conakry; Agence Nationale de Sécurité Sanitaire de Guinée; University of Sciences, Techniques and Technologies of Bamako (USTTB); Faculty of Health Sciences and Technology, Gamal Abdel Nasser University of Conakry

**Keywords:** Resources, associated factors, quality, management, epidemic-prone diseases, Guinea

## Abstract

**Objective:**

This study aimed to describe the evolution of resources and to identify the factors associated with the quality of care for epidemic-prone diseases in Guinea.

**Method:**

A descriptive cross-sectional study focused on the management systems for epidemic-prone diseases in Guinea. Data were collected using structured questionnaires. The analysis of factors associated with the quality of care was conducted using logistic regression.

**Results:**

The evolution of management systems varied across regions. Overall, at the national level, before the Ebola outbreak, the evolution of management systems was low, with an overall score of 12%. During the Ebola virus crisis, the evolution of the systems was average, reaching 73%, and after the outbreak, it remained average at 77%. The quality of care was predominantly rated as good, accounting for 62.5%. The main factors associated with better quality of care included the presence of a patient diagnostic mechanism (aOR = 8; 95% CI [2.02–19.45]; p = 0.000), the availability of treatment protocols (aOR = 9.44; 95% CI [1.02–12.02]; p = 0.000), the presence of isolation sites (aOR = 4.23; 95% CI [1.61–9.66]; p = 0.000), the coordination of care (aOR = 8.5; 95% CI [1.22–12.00]; p = 0.002), and the availability of care teams (aOR = 8.5; 95% CI [1.22–12.00]; p = 0.001).

**Conclusion:**

This study highlights significant progress in resources for the management of epidemic-prone diseases in Guinea during and after the Ebola crisis. Key factors such as diagnostic tools, treatment protocols, the availability of isolation sites, care coordination, and the presence of care teams were essential for success. The overall improvement reflects strengthened capacity and preparedness in Guinea, although challenges remain, particularly in terms of financial resources.

## INTRODUCTION

Epidemic diseases are infections that can affect multiple people simultaneously and spread from one person to another within a given locality [1].

The West African region has been the site of several epidemics and outbreaks of infectious diseases, leading to high morbidity and mortality in certain West African countries, with negative consequences for the health systems of these countries [2] . Among these, we can mention anthrax in Guinea; cholera in Benin, Liberia, Nigeria, and Sierra Leone; dengue fever in Benin and Côte d’Ivoire; yellow fever in Nigeria; meningitis in Ghana, Niger, and Togo; and measles in Guinea, Niger, and Sierra Leone. Lassa fever is endemic to West Africa and has been reported in Sierra Leone, Guinea, Liberia, and Nigeria [3].

Guinea faced several health emergencies, the most notable being the Ebola virus disease (EVD) outbreak between 2014 and 2016. Prior to this crisis, the Guinean health system exhibited significant weaknesses, including limited geographic coverage due to inadequate infrastructure and equipment, an overall fragile state, low access to healthcare services (38.9%) and low utilization of these services (18.6%) [4]. The Ebola experience revealed shortcomings in terms of human resources, medical staff training, adequate healthcare infrastructure, and access to care, particularly in rural areas [5]. These structural weaknesses were exacerbated during the epidemic by major challenges, such as a shortage of qualified human resources, difficulties in the supply of essential inputs, limited coordination among stakeholders, and delays in diagnosis and case management. In response to this situation, substantial measures were implemented to control the epidemic, including community mobilization strategies, the establishment of community transit centers (CTCom) and Ebola treatment centers (ETC), the capacity building of healthcare personnel to improve early case detection, the systematic isolation of patients in the ETC, and the strengthening of infection prevention and control measures in healthcare facilities [6–8]. Despite these efforts, several challenges persisted, particularly related to sustainable financing, logistics, community adherence, and the continuity of essential health services. During the post-Ebola recovery phase, Guinea progressively strengthened its health system by establishing coordination, surveillance, diagnostic, and care structures at all levels of the health system. These initiatives include the creation of the National Health Security Agency, the deployment of regional and prefectural epidemic alert and response teams, the establishment of decentralized public health emergency operations centers, the development of hemorrhagic fever diagnostic laboratories, and the implementation of epidemic treatment centers [9,10]. However, challenges remain, particularly regarding the sustainability of financing, infrastructure maintenance, the continuous availability of inputs, and the equitable distribution of resources across regions. Currently, epidemic treatment centers are staffed with regularly trained personnel, ensuring the management of suspected or confirmed cases of epidemic-prone diseases or priority zoonoses in accordance with established national protocols [8]. The quality of management of epidemic-prone diseases depends on many similar factors [11–14]. However, although several interventions have been implemented during epidemics (notably Ebola), systematic analyses examining both the evolution of health system resources (human, material, financial, informational, and temporal) and the determinants of care quality in the context of multiple and simultaneous epidemics are lacking. Our study aims to describe the evolution of resources and to examine the factors associated with the quality of care for epidemic-prone diseases in Guinea in the context of multiple and simultaneous epidemics.

### Research question:

How does the evolution of health system resources influence the quality of care for epidemic-prone diseases in Guinea in the context of multiple epidemics?

### Research hypothesis:

Improved availability and organization of health system resources are associated with better quality of care for epidemic-prone diseases in Guinea.

## STUDY METHODS

### Study Framework

The Republic of Guinea is a coastal country located in the western part of the African continent, halfway between the Equator and the Tropic of Cancer (7°30’ and 12°30’ north latitude and 8° and 15° west longitude, respectively). Covering an area of 245,857 km^2^, it bordered west by Guinea-Bissau and the Atlantic Ocean, north by Senegal and Mali, east by Côte d’Ivoire, and south by Sierra Leone and Liberia [15]. From a geoecological perspective, Guinea is divided into four distinct natural regions that are internally homogeneous: Guinea Maritime, Middle Guinea, Upper Guinea, and Forest Guinea [16,17]. Guinea is distinguished by its natural environment, characterized by climatic contrasts, mountainous barriers, and the orientation of the reliefs, creating regional specificities. Administratively, it is divided into eight regions, 33 prefectures, and 38 urban communities, five of which are in Conakry, and 303 rural communities [16,17]. It is among the countries in West Africa most severely affected by the Ebola virus disease epidemic [18].

### Study site

The study site will encompass all three national directorates, as well as the thirty-eight (38) health districts of Guinea. Eight (08) regional health inspections, thirty-eight (38) prefectural health directorates, and thirty-eight (38) epidemic treatment centers play crucial roles in the epidemic response in the Republic of Guinea.

#### Study Type:

A descriptive cross-sectional study with an analytical aim was conducted from April 2024 to October 2025. A mixed-methods approach was adopted because of the diversity of information sources.

### The target and source populations

It consists of all the actors involved in the epidemic response in Guinea, including those from the technical departments of the Ministry of Health, regional health inspections, the prefectural health directorates, and epidemic treatment centers.

#### • Inclusion criteria:

Actors directly involved in epidemic response across the eight administrative regions of Guinea agreed to participate in the study.

#### • Exclusion criteria:

Actors who were not involved in the epidemic response, as well as those who did not consent to participate or who provided incomplete data, were excluded.

### Sampling

We used a probabilistic sampling method based on a two-stage stratified sampling approach, in which regions constitute the primary statistical units and epidemic response actors constitute the secondary statistical units. The selection of response actors in each district was based on the development of a comprehensive sampling frame, established with the support of operational teams already mobilized across the eight health regions. This sampling frame included, in particular, Prefectural Health Directors, disease control physicians, planning, training and research officers, staff of epidemic treatment centers, data managers, logisticians, laboratory managers, members of response committees, communication officers, hospital directors, Public Health Emergency Operations Center staff, case management officers, and technical and financial partners.

We used the Schwartz formula to determine the minimum sample size.


$$n={\mathrm{Z\alpha}}^{2}\left(p\times q\right)/i^{2}$$

With:

- Zα is the standard normal deviate for a 95% confidence level, equal to 1.96
- p = expected proportion 50% = 0.5
- q = 1 − p = 0.5
- d = desired margin of error = 5% = 0.05

Numerical application: n = minimum sample size = **400 participants.**

### Study variables

- **Identical variables related to the evolution of resources before, during and after the Ebola virus disease (EVD):** 1. Human Resources: Availability of trained health workers, availability of sufficient human resources for case management, existence of a supervision plan for case management actors, and existence of a coordination mechanism for case management activities; 2. Material Resources: availability of functional isolation sites, availability of medicines and inputs for case management, availability of diagnostic laboratories, availability of mobile logistics for case management, availability of a computer at the case management site, and availability of a printer at the case management site; 3. Financial Resources: availability of funding for case management and provision of food within case management; 4. Informational Resources: availability of case management protocols, availability of case definition forms, existence of a case reporting system, availability of a case transfer protocol, and existence of mechanisms for continuity of care for patients with complications; 5. Temporal Resources: prompt case management.
- **Dependent variable:** The dependent variable was the quality of management of epidemic-prone diseases. It was a dichotomous variable and was assessed on the basis of the presence or absence of appropriate management strategies for these diseases. The criteria for evaluating the quality of management of epidemic-prone diseases were as follows [19]: 1. Early diagnosis of patients, 2. Timeliness of patient care, 3. Early isolation of patients, 4. Regular organization of care coordination meetings, 5. Availability of staff involved in case management, 6. Safety of patient care, 7. Safe treatments were administered in accordance with the protocol. The evaluation criteria were as follows:
  - The identification of at least six (6) quality criteria, including the administration of safe treatments in accordance with protocols, was considered good-quality care (score = 1);
  - The identification of fewer than six (6) quality criteria, with the absence of the administration of safe treatments in accordance with protocols, was considered poor-quality care (score = 0).
- **Independent variables:** Age, marital status, level of education, position held, place of residence, response commission, presence of a functional isolation site, presence of staff trained in the management of epidemic-prone diseases (EPDs), availability of medications and care inputs, presence of a functional laboratory for EPD diagnosis, availability of diagnostic tests, presence of functional mobile logistics, presence of a dedicated fund for care during epidemics, availability of an EPD management guide, presence of a functional borehole at the isolation site, support from technical and financial partners, availability of infection prevention and control kits at the isolation site, availability of a waste management area at the isolation site, availability of a care management protocol, existence of a psychosocial care mechanism, availability of an infection prevention and control manual, availability of an EPD management guide, existence of an EPD diagnostic protocol, availability of a continuity of care plan for complicated cases, existence of a supervision plan for care providers, availability of a medication and input supply plan, existence of an isolation site rehabilitation plan, availability of a patient transfer protocol, existence of a case reporting mechanism, and presence of a coordination mechanism for EPD care activities during outbreaks.

### Data collection techniques and tools

Data collection was primarily based on individual interviews conducted with epidemic response actors. It was complemented by secondary data from direct observations and a literature review.

The main data collection tools included interview and observation guides, a structured questionnaire, and a data extraction form. All of these tools were pretested to ensure their clarity, relevance, and consistency before deployment in the field. Data collection was carried out with the support of health workers from the National Health Security Agency, who were previously trained to ensure standardized procedures and the quality of the data collected.

### Data validity

Several measures were implemented to ensure the validity and reliability of the data collected in this study.

- First, the data collection tools (questionnaire, interview and observation grids, and data extraction form) were pretested in the meeting room of the National Health Security Agency. This pretest helped assess the clarity, relevance, and consistency of the questions and allowed for necessary adjustments before field deployment;
- Second, in-depth training was provided to the surveyors to standardize the data collection procedures. The training covered understanding of the tools, interview techniques, objective observation, and management of potential biases, including information bias, recall bias, and subjectivity bias;
- In addition, data triangulation was applied by cross-checking information from multiple sources, including participant interviews, direct field observations, and document reviews (reports, registers, and other available records). This approach enhanced the credibility and robustness of the collected data;
- Finally, data verification and validation procedures were implemented throughout the data collection process. Field supervisors conducted daily checks to identify and correct inconsistencies or missing data. Further quality control was carried out during data entry and cleaning to ensure consistency and reliability for analysis.

### Data Processing and Analysis

- Data were entered and managed using KoboToolbox and exported for analysis. Statistical analyses were primarily performed using Stata (version 13). Descriptive analysis was conducted to summarize the characteristics of the study population and the distribution of the variables. The descriptive analysis involved describing the study sample through a detailed description of the variables. Qualitative variables are described in terms of absolute and relative frequency, whereas quantitative variables are described as the mean ± standard deviation. Aggregated data (regional and national means) were used exclusively to describe the evolution of resources, whereas analytical analyses, particularly logistic regression, were performed using individual-level data.
- Analysis of the evolution of resources for the management of epidemic-prone diseases focused on changes in resources before, during, and after the Ebola virus disease outbreak in Guinea. This analysis was conducted in two stages: the first focused on the evolution of the systems at the regional level, covering a total of eight (8) regions, and the second consisted of an overall analysis at the national level. To interpret the results regarding changes in resources, the WHO criteria for classifying health system resources were used. [20–22]. The regional evolution of resources for the management of epidemic-prone diseases was estimated on the basis of the percentages of resources calculated for each of the eight administrative regions. These percentages were obtained by averaging the resource indicators observed at the health district level within each region. The overall evolution of resources at the national level was determined by aggregating regional results through the average of the percentages across the eight administrative regions. Finally, the performance levels of the different resources were assessed using the measurement scale proposed by Varkevisser [23]. This evaluation allowed the results to be classified into the following categories [23]:
  - “Good evolution”: for a percentage between [80% and 100%];
  - “Average evolution”: for a percentage between [60% and 80%];
  - “Poor evolution”: for a percentage between [0% and 60%].
- Factors associated with the quality of care for epidemic-prone diseases were identified on the basis of data from the year 2025. The determinants were analyzed using a two-step approach:
  - First, a univariate analysis was performed by cross-tabulating the dependent variable with the independent variables;
  - Multivariate analysis was subsequently conducted using stepwise backward multiple logistic regression. At this stage,all statistically significant variables at the 20% threshold in the univariate analysis were included in the initial model. Variables with a p value lower than 20% were retained. Multivariate analysis was then performed to progressively eliminate variables with the highest p values using a stepwise backward approach until the final model was obtained. Variables associated with a 5% threshold were retained in the final model;
  - To assess the goodness of fit of the final model, the Hosmer–Lemeshow test was performed, with a p value greaterthan 5%.

### Ethical considerations

We obtained approval from the National Ethics Committee for Health Research (NECHR) (No. 122/NECHR/24), as well as authorization for data collection from the General Directorate of the National Health Security Agency. An information sheet was provided to the participants, and informed consent was obtained prior to the administration of the questionnaire and the individual interviews. Anonymity and confidentiality were ensured throughout the data collection and analysis process.

## RESULTS

### Sociodemographic and professional characteristics

A total of 400 epidemic response actors in Guinea were surveyed, covering eight (8) administrative regions. Among the participants, the majority were female, accounting for 75.4%, whereas 24.6% were male, accounting for 3.23 women for every man. The average age of the participants was 52.33 ± 6.23 years, with the following distribution: 46% were under 40 years old, 54% were between 40 and 60 years old, and 3% were over 60 years old. In terms of education level, the vast majority of the participants (91.77%) held a university degree, whereas 8.23% had a secondary school degree.

### Evolution of resources for the management of epidemic-prone diseases in Guinea

#### Evolution of resources for the management of epidemic-prone diseases at the level of Guinea’s administrative regions

- **Human resources**: Human resources were low before the Ebola virus disease (EVD) outbreak across all regions, with levels ranging from 11% to 12% in Conakry (12%), Kindia (11%), Boké (12%), Mamou (12%), Faranah (11%), Labé (11%), Kankan (11%), and N’Zérékoré (11%). During the outbreak, a marked improvement was observed in all regions, reaching 75% in Conakry, Kindia, Boké, Mamou, Kankan, and N’Zérékoré, 74% in Labé, and 73% in Faranah. After the outbreak, this progress was sustained, with high levels ranging between 77% and 79%, notably 79% in most regions and 77% in Faranah. Overall, human resources significantly and consistently improved across all the regions.
- **Material resources and supplies**: Material resources were also limited before the outbreak, with levels ranging between 8% and 9% across all regions. During the outbreak, a substantial increase was observed, with levels reaching 73% in Conakry and Kindia, 72% in most other regions, and 71% in Faranah. After the outbreak, material resources remained at high levels, ranging between 77% and 79%, with 79% in several regions (Kindia, Mamou, Labé, Kankan, N’Zérékoré) and 77% in Faranah. This trend reflects a significant and sustained strengthening of equipment and essential supplies for care.
- **Financial resources**: Financial resources were low before the outbreak in all regions (9% to 10%). During the crisis, they increased substantially, reaching 72% in Conakry, Kindia, Boké, Mamou, and N’Zérékoré; 71% in Labé and Kankan; and 70% in Faranah. However, after the outbreak, a notable decline was observed across all regions, with levels ranging between 49% and 51% (49% in Conakry, Mamou, and Faranah; 50% in Kindia, Boké, and Kankan; and 51% in Labé and N’Zérékoré). Unlike other resources, financial resources showed a postcrisis decline, highlighting challenges related to the sustainability of funding.
- **Informational resources**: Informational resources were low before the outbreak, ranging between 17% and 19% across regions. During the outbreak, they improved significantly, reaching 80% in Conakry; 79% in several regions (Kindia, Boké, Mamou, Kankan, N’Zérékoré); and 78% in Faranah and Labé. After the outbreak, these resources reached very high levels, ranging between 87% and 89%, with 89% in most regions, 88% in Labé, and 87% in Faranah. These findings indicate a sustained strengthening of information systems, protocols, and management tools.

### Temporal resources (response timeliness)

Temporal resources, reflecting response timeliness, were low before the outbreak across all regions (15% to 16%). During the outbreak, significant improvements were observed, with 67% in Conakry, Kindia, Boké, Kankan, and N’Zérékoré; 66% in Mamou and Labé; and 65% in Faranah. After the outbreak, these resources reached high levels, ranging between 85% and 87%, with 87% in several regions, 86% in Labé and N’Zérékoré, and 85% in Faranah. This reflects a sustained improvement in the responsiveness of the health system. (See Table I).

### Overall evolution of resources for the management of epidemic-prone diseases at the national level in Guinea.

The overall analysis of resources for the management of epidemic-prone diseases in Guinea revealed significant improvements across the preepidemic, epidemic, and postepidemic periods. Before the Ebola virus disease (EVD) outbreak, all categories of resources were at low levels, with 12% for human resources, 8% for material resources, 9% for financial resources, 18% for informational resources, and 15% for temporal resources, reflecting structural weaknesses in the health system. During the outbreak, a substantial improvement was observed across all resource categories, reaching 74% for human resources, 72% for material resources, 71% for financial resources, 79% for informational resources, and 67% for temporal resources, indicating a strong mobilization of health system capacities in response to the crisis. After the outbreak, most resources continued to improve or were maintained at high levels, particularly human resources (79%), material resources (78%), informational resources (89%), and temporal resources (87%). In contrast, financial resources declined to 50%, highlighting challenges related to the sustainability of funding in the postcrisis period. Overall, resource levels increased from 12% before the outbreak to 73% during the epidemic and stabilized at 77% after, with the overall assessment improving from poor to average, indicating a substantial—although still incomplete—strengthening of the health system’s capacity to manage epidemic-prone diseases in Guinea (see Table II).

### Factors associated with the quality of care for epidemic-prone diseases in Guinea

**Figure 2:**
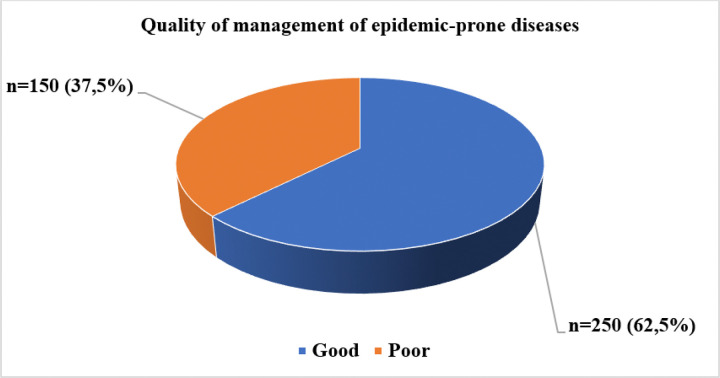
Management quality of epidemic-prone diseases in Guinea in 2025.

The quality of management of epidemic-prone diseases in Guinea during this study was predominantly rated as good, accounting for 62,5%, compared to 37,5% for poor quality (see Fig. 2).

### Factors associated with univariate analysis

According to the univariate analysis, several factors were significantly associated with the quality of care for epidemic-prone diseases. The presence of a patient diagnostic mechanism was strongly associated with better quality of care (OR = 24.13; 95% CI [5.21–28.32]; p = 0.000). This finding indicates that facilities with a diagnostic mechanism had approximately 24 times higher odds of providing quality care than those without such a mechanism did. The availability of treatment protocols was also significantly associated with quality of care (OR = 12.63; 95% CI [2.55–16.24]; p = 0.000), suggesting that facilities using standardized protocols had approximately 12 times higher odds of delivering quality care. The presence of isolation sites was another important factor (OR = 9.23; 95% CI [2.55–15.50]; p = 0.000), indicating that the presence of isolation facilities increased the odds of quality care by approximately ninefold. Coordination of care was significantly associated with better outcomes (OR = 10.77; 95% CI [2.51–16.83]; p = 0.000), as was the availability of care teams (OR = 10.77; 95% CI [2.51–16.83]; p = 0.000), meaning that these factors increased the likelihood of quality care by approximately ten times. In contrast, psychosocial care for patients, although positively associated (OR = 1.96; 95% CI [0.81–19]), was not statistically significant (p = 0.056). This suggests a potential positive effect (nearly twofold higher odds) but with uncertainty. Finally, funding for care was significantly inversely associated (OR = 0.34; 95% CI [0.15–0.74]; p = 0.007). This finding indicates that facilities with funding had approximately 66% lower odds of poor-quality care, highlighting the protective role of financial support. (See Table III).

### Factors associated with multivariate analysis

In the multivariate analysis, several factors remained independently associated with the quality of care for epidemic-prone diseases in Guinea. The presence of a patient diagnostic mechanism was strongly associated with better quality of care (aOR = 8; 95% CI [2.02–19.45]; p = 0.000). This means that facilities with a diagnostic mechanism had approximately 8 times higher odds of providing quality care than those without such a mechanism did. The availability of treatment protocols was also significantly associated with quality of care (aOR = 9.44; 95% CI [1.02–12.02]; p = 0.000), indicating that facilities applying standardized protocols had approximately 9 times higher odds of delivering quality care. The presence of isolation sites was an independently associated factor (aOR = 4.23; 95% CI [1.61–9.66]; p = 0.000), meaning that the presence of isolation sites increased the odds of adequate care by approximately fourfold. Coordination of care remained a key determinant (aOR = 8.5; 95% CI [1.22–12.00]; p = 0.002), reflecting an approximately eightfold increase in the odds of providing quality care when activities are well coordinated. Similarly, the availability of care teams was significantly associated with the quality of care (aOR = 8.5; 95% CI [1.22–12.00]; p = 0.001), indicating that the presence of functional teams increases the likelihood of quality care by approximately eight times. (See Table III).

## DISCUSSION

### Evolution of resources for the management of epidemic-prone diseases

The findings of this study show a significant improvement in the resources mobilized for the management of epidemic-prone diseases in Guinea, with an overall progression from low levels before the outbreak to moderate levels during and after the crisis. This improvement concerns mainly human, material, informational, and temporal resources, while financial resources declined during the postepidemic period. Before the outbreak, the low levels observed across all resource categories (human, material, financial, informational, and temporal) reflected structural weaknesses in the Guinean health system, characterized by limited infrastructure, a shortage of qualified human resources, weak information systems, and insufficient coordination mechanisms. These constraints contributed to the country’s vulnerability to major health crises, such as Ebola virus disease (EVD). During the outbreak, significant and concurrent improvements across all categories of resources were observed, reflecting an exceptional mobilization of national and international capacities. This dynamic was particularly evident in material resources, with the construction of Ebola Treatment Centers (ETC), the establishment of community transit centers (CTCom), and the deployment of temporary care facilities. The improvement in human resources reflects sustained efforts in training and staff deployment. Moreover, progress in material and informational resources highlights increased investments in equipment, essential supplies, surveillance systems, and clinical management protocols. Furthermore, the strengthening of temporal resources indicates a notable increase in system responsiveness, particularly in terms of case detection, reporting, and timely patient management. This evolution can largely be attributed to strengthened health governance at the central level, marked by the establishment of key institutional mechanisms such as the national Ebola response coordination and prefectural coordination units, as well as continued support from technical and financial partners. In the postepidemic period, our findings show a consolidation of gains across most resource categories, particularly human, material, informational, and temporal resources, which reached high levels. This suggests that the Ebola crisis acted as a catalyst for structural and organizational reforms, contributing to improved health system resilience. These post-EVD advances were accompanied by the establishment and strengthening of key national structures, including the construction of thirty-eight (38) epidemic treatment centers (CTEPIs) across all prefectures, whether hospital-based or standalone. They also include the strengthening of the National Health Security Agency, the establishment of decentralized public health emergency operations centers, and the development of integrated disease surveillance systems. These mechanisms have improved the coordination of interventions and standardized clinical management protocols for major epidemic-prone diseases (including Ebola, COVID-19, Lassa fever, mpox, measles, cholera, Marburg, and yellow fever) and enhanced information flow across all levels of the health system. Furthermore, the development of normative frameworks, national operational guidelines for epidemic treatment centers, clinical management protocols for epidemic-prone diseases and priority zoonoses, along with strengthened coordination mechanisms, has contributed to harmonizing practices and reinforcing response governance. This positive trajectory contrasts with the significant decline in financial resources observed after the outbreak, highlighting a persistent vulnerability related to reliance on emergency funding and the lack of sustainable domestic financing mechanisms. This poses a major challenge to the sustainability of achievements and long-term preparedness. A study conducted in Guinea by KPAMY DO and coll. [24] reported a lack of funding for the management of epidemic-prone diseases in Guinea. The same finding regarding insufficient funding was highlighted in a study on the success factors of the response to the Mpox outbreak in Guinea [25]. Although the trends were generally consistent across regions, some disparities remain, suggesting inequalities in resource distribution and the implementation of interventions. These gaps underscore the need for strengthened national-level strategic leadership to ensure equitable resource allocation and improved coordination across health system levels. Moreover, these findings confirm that epidemics can serve as opportunities for health system transformation, particularly in resource-limited settings, by fostering institutional reforms, capacity building, and improvements in information and coordination systems [26].

### Factors associated with the quality of care for epidemic-prone diseases in Guinea

The results of the univariate and multivariate analyses revealed that several factors play a significant role in improving the quality of care for EPD patients. The quality of care for epidemic-prone diseases in Guinea was largely rated as good, indicating the effective performance of the systems for managing EPD in the country [24]. Our results revealed that the factors associated with the quality of care for epidemic-prone diseases (EPDs) in Guinea include the presence of a diagnostic mechanism, the availability of treatment protocols, the existence of isolation sites, the coordination of care, and the availability of care teams. One of the most significant findings of this study is the strong association between the presence of a diagnostic mechanism for epidemic-prone diseases and their good quality of care. It is evident that the establishment of an effective mechanism for diagnosing epidemic-prone diseases is essential for the rapid and appropriate management of epidemics. This association underscores the importance of a robust diagnostic system to ensure effective management of epidemic-prone diseases. The availability of treatment protocols for epidemic-prone diseases is also an associated factor in the quality of care. This suggests that institutions following standardized protocols are six times more likely to provide quality care. These findings highlight the importance of having clear and well-established guidelines for patient management, especially during epidemics. The presence of isolation sites for epidemic-prone diseases was another associated factor. These results emphasize the importance of isolating suspected or confirmed cases of EPD promptly to limit the spread of disease. Patient isolation is a key element in the management of communicable diseases and is strongly correlated with the quality of care provided. DO. KPAMY et al. demonstrated, in their evaluation of the management systems for epidemic-prone diseases, that the functional availability of diagnostic laboratories for epidemic diseases and epidemic treatment centers were key strengths [24]. Coordination of care is also crucial. These results indicate that coordinating efforts among various actors, whether care teams, local authorities, or international partners, plays a major role in managing epidemics. Good coordination allows for effective resource allocation, better communication, and a faster response. Since the Ebola outbreak, coordination among health actors has proven essential for effective crisis management. This coordination relies on the regular organization of meetings to monitor activities, manage crises, or coordinate responses [27].

The factor most significantly associated with better quality of care in our study was the availability of care teams. These findings demonstrate that the size and competence of care teams are essential determinants of the quality of care. Continuous training of healthcare staff, particularly in epidemic treatment centers, is therefore a key factor in ensuring an effective response to health crises. In the Democratic Republic of Congo and Sierra Leone, during the Ebola outbreak, the lack of qualified personnel hindered response efforts [28, 29]. In contrast, during the tenth and twelfth Ebola outbreaks in the DRC, the involvement of local actors enhanced the effectiveness of interventions [30]. The psychosocial care of patients, while positive, did not show a statistically significant link with the quality of care. This suggests that although this approach is important for patient well-being, it may not have as direct an impact on the quality of physical care, which likely requires greater attention in future strategies. The financing of care was inversely associated with the quality of care. This result highlights a major issue: the lack of dedicated funding for epidemic management can significantly undermine the quality of care. Access to financial resources is essential for implementing prevention and treatment measures, emphasizing the need for sustainable and responsive funding mechanisms. DO. KPAMY et al. also highlighted the lack of funding for management systems for epidemic-prone diseases in Guinea [24]. In a study of the success factors in the response to the Mpox epidemic in Guinea, financing was not identified as an associated factor [25].

### Implications for Future Epidemic Responses

The results of this study highlight the importance of maintaining a balanced approach to resource allocation for epidemic management. While the massive mobilization of resources during the crisis was necessary and effective, future responses should focus on sustaining the progress made in terms of human, material, and informational resources. The reduction in financial resources after the crisis indicates a potential vulnerability in the country’s ability to respond effectively to future epidemics [24, 25, 31]. It is therefore essential to establish sustainable financing mechanisms that can support long-term preparedness and response. As in other countries in the African subregion, future epidemics must be managed equitably through the establishment or reactivation of rapid response teams (RRTs), with the support of the Guinean government as well as technical and financial partners [32–35].

### Study limitations

This study presents certain limitations that should be taken into account when the results are interpreted.

- Owing to operational constraints related to the implementation of field activities, some of the initially targeted participants were not available for interviews or to respond to the questionnaires, which may have affected the participation rate;
- The use of self-reported data from response actors may introduce information bias, particularly social desirability bias, as participants may tend to overestimate the performance of epidemic management systems.

Despite these limitations, several measures were implemented to strengthen data validity, including data triangulation (interviews, observations, and document review), pretesting of data collection tools, and training of surveyors.

## CONCLUSION

This study highlights a significant improvement in resources for the management of epidemic-prone diseases in Guinea during and after the Ebola outbreak, reflecting the overall strengthening of the health system. It also identifies key factors associated with the quality of care, including the availability of diagnostic mechanisms, treatment protocols, isolation sites, effective coordination, and care teams. However, the decline in financial resources after the crisis represents a major limitation to the sustainability of these gains. In addition, regional disparities persist, underscoring the need for stronger strategic leadership.

Sustained strengthening of resources, including the reactivation of rapid response teams (RRTs), consolidation of factors associated with quality of care, and the establishment of sustainable financing mechanisms, are essential to enhancing the resilience of the health system and ensuring effective responses to future epidemics in Guinea.

## Figures and Tables

**Figure 1:**
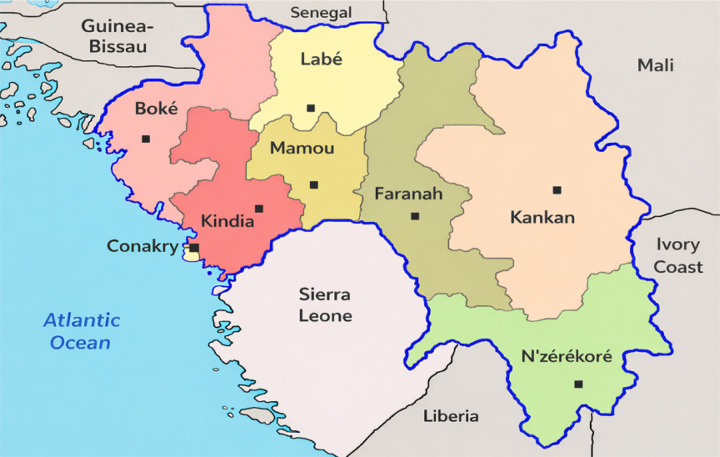
Health Map of Guinea According to the Eight (8) Administrative Regions (Source: https://www.axl.cefan.ulaval.ca/afrique/guinee_fr-carteregionale.htm).

**Table I:** Evolution of resources for the management of epidemic-prone diseases at the level of each administrative region of Guinea before, during, and after the Ebola virus disease outbreak.

Resources/Region	Before EVD	During EVD	After EVD
Human Resources			
Conakry (n = 96)	12/96 (12%)	72/96 (75%)	76/96 (79%)
Kindia (n = 49)	5/49 (11%)	37/49 (75%)	39/49 (79%)
Mamou (n = 24)	3/24 (12%)	18/24 (75%)	19/24 (79%)
Faranah (n = 23)	3/23 (11%)	17/23 (73%)	18/23 (77%)
Labé (n = 19)	2/19 (11%)	14/19 (74%)	15/19 (79%)
Boké (n = 23)	3/23 (12%)	17/23 (75%)	18/23 (79%)
Kankan (n = 79)	9/79 (11%)	59/79 (75%)	62/79 (79%)
N’Zérékoré (n = 87)	10/87 (11%)	65/87 (75%)	69//87 (79%)
Overall	**47/400 (12%)**	**299/400 (74%)**	**316/400 (79%)**
Assessment	**Poor**	**Average**	**Average**
Material Resources			
Conakry (n = 96)	9/96 (9%)	70/96 (73%)	75/96 (78%)
Kindia (n = 49)	4/49 (8%)	36/49 (73%)	39/49 (79%)
Mamou (n = 24)	2/24 (8%)	17/24 (72%)	19/24 (79%)
Faranah (n = 23)	2/23 (9%)	16/23 (71%)	18/23 (77%)
Labé (n = 19)	2/19 (8%)	14/19 (72%)	15/19 (79%)
Boké (n = 23)	2/23 (8%)	17/23 (72%)	18/23 (78%)
Kankan (n = 79)	6/79 (8%)	57/79 (72%)	62/79 (79%)
N’Zérékoré (n = 87)	7/87 (8%)	63/87 (72%)	69/87 (79%)
Overall	**34/400 (8%)**	**290/400 (72%)**	**315/400 (78%)**
Assessment	**Poor**	**Average**	**Average**
Financial Resources			
Conakry (n = 96)	10/96 (10%)	69/96 (72%)	47/96 (49%)
Kindia (n = 49)	4/49 (9%)	35/49 (72%)	25/49 (50%)
Mamou (n = 24)	2/24 (9%)	17/24 (72%)	12/24 (49%)
Faranah (n = 23)	2/23 (9%)	16/23 (70%)	11/23 (49%)
Labé (n = 19)	2/19 (9%)	13/19 (71%)	10/19 (51%)
Boké (n = 23)	2/23 (9%)	17/23 (72%)	12/23 (50%)
Kankan (n = 79)	7/79 (9%)	56/79 (71%)	40/79 (50%)
N’Zérékoré (n = 87)	8/87 (9%)	63/87 (72%)	44/87 (51%)
Overall	**37/400 (9%)**	**286/400 (71%)**	**201/400 (50%)**
Assessment	**Poor**	**Average**	**Poor**
Informational Resources			
Conakry (n = 96)	18/96 (19%)	77/96 (80%)	85/96 (89%)
Kindia (n = 49)	8/49 (17%)	39/49 (79%)	44/49 (89%)
Mamou (n = 24)	4/24 (18%)	19/24 (79%)	21/24 (89%)
Faranah (n = 23)	4/23 (18%)	18/23 (78%)	20/23 (87%)
Labé (n = 19)	3/19 (17%)	15/19 (78%)	17/19 (88%)
Boké (n = 23)	4/23 (17%)	18/23 (79%)	20/23 (89%)
Kankan (n = 79)	13/79 (17%)	62/79 (79%)	70/79 (89%)
N’Zérékoré (n = 87)	15/87 (17%)	69/87 (79%)	77/87 (89%)
Overall	**69/400 (18%)**	**317/400 (79%)**	**355/400 (89%)**
Assessment	**Poor**	**Average**	**Average**
Temporal Resources			
Conakry (n = 96)	15/96 (16%)	64/96 (67%)	84/96 (87%)
Kindia (n = 49)	7/49 (15%)	33/49 (67%)	43/49 (87%)
Mamou (n = 24)	4/24 (15%)	16/24 (66%)	21/24 (87%)
Faranah (n = 23)	4/23 (16%)	15/23 (65%)	20/23 (85%)
Labé (n = 19)	3/19 (16%)	13/19 (66%)	16/19 (86%)
Boké (n = 23)	4/23 (15%)	15/23 (67%)	20/23 (87%)
Kankan (n = 79)	12/79 (15%)	53/79 (67%)	69/79 (87%)
N’Zérékoré (n = 87)	13/87 (15%)	58/87 (67%)	75/87 (86%)
Overall	**62/400 (15%)**	**267/400 (67%)**	**348/400 (87%)**
Assessment	**Poor**	**Average**	**Average**

**Table II:** Overall evolution of resources for the management of epidemic-prone diseases at the national level in Guinea before, during, and after the Ebola virus disease outbreak.

National level Resources	Before EVD N = 400	During EVD N = 400	After EVD N = 400
**Human Resources**	47 (12%)	299 (74%)	316 (79%)
**Material Resources**	34 (8%)	290 (72%)	315 (78%)
**Financial Resources**	37 (9%)	286 (71%)	201 (50%)
**Informational Resources**	69 (18%)	317 (79%)	355 (89%)
**Temporal Resources**	62 (15%)	267 (67%)	348 (87%)
**Overall Evolution of Resources**	**49,8 (12%)**	**291,8 (73%)**	**307 (77%)**
**Assessment of the evolution**	**Poor**	**Average**	**Average**

**Table III:** Factors associated with the quality of management of epidemic-prone diseases in Guinea according to univariate analysis

Factors associeted	Frequency (n)	P value	OR	95% CI
**Presence of a patient diagnostic mechanism**				
No	88	-	1	-
Yes	312	0,000	24.13	[5.21–28.32]
**Availability of treatment protocols for patients**				
No	48	-	1	-
Yes	352	0,000	12.63	[2.55–16.24]
**Existence of isolation sites for patients**				
No	87	-	1	-
Yes	313	0,000	9.23	[2.55–15.50]
**Coordination of care**				
No	85	-	1	-
Yes	315	0,000	10.77	[2.51–16.83]
**Availability of care teams**				
No	85	-	1	-
Yes	315	0,000	10.77	[2.51–16.83]
**Psychosocial care for patients**				
No	40	-	1	-
Yes	360	0,056	1.96	[0.81–19]
**Funding for care**				
No	360	-	1	-
Yes	40	0,007	0.34	[0.15–0.74]

**Table III:** Factors associated with the quality of care for epidemic-prone diseases in Guinea according to multivariate analysis

Factors associated	Effectif (n)	P- value	aOR	IC 95%
**Presence of a patient diagnostic mechanism**				
No	88	-	1	-
Yes	312	0,000	08	[2,02–19,45]
**Availability of treatment protocols for patients**				
No	48	-	1	-
Yes	352	0,000	9.44	[1.02–12.02]
**Existence of isolation sites for patients**				
No	87	-	1	-
Yes	313	0,000	4.23	[1.61–9.66]
**Coordination of care**				
No	85	-	1	-
Yes	315	0,002	8,5	[1.22–12.00]
**Availability of care teams**				
No	85	-	1	-
Yes	315	0,001	8,5	[1.22–12.00]

## Data Availability

The data generated and/or analyzed during this study are available from the corresponding author upon reasonable request.
